# Outcomes of Bovine Jugular Vein Versus Porcine Valved Conduits for Right Ventricle to Pulmonary Artery Connection

**DOI:** 10.1007/s00246-025-03885-7

**Published:** 2025-05-10

**Authors:** Bahar Temur, Ibrahim Gokce, Yakup Tire, Zeynep Sila Ozcan, Selim Aydin, Tugcin Bora Polat, Ersin Erek

**Affiliations:** 1 Department of Cardiovascular Surgery, Acibadem Mehmet Ali Aydinlar University School of Medicine, Istanbul, Turkey; 2 Acibadem Mehmet Ali Aydinlar University School of Medicine, Istanbul, Turkey; 3 Department of Cardiovascular Surgery, Acibadem Atakent Hospital, Istanbul, Turkey; 4 Department of Pediatric Cardiology, Acibadem Atakent Hospital, Istanbul, Turkey; 5 Department of Pediatric Cardiovascular Surgery, Acibadem Mehmet Ali Aydinlar University School of Medicine, Istanbul, Turkey

**Keywords:** Bovine jugular vein conduit, Congenital heart disease, Pulmonary atresia, Ventricular septal defect, Right ventricle outflow tract reconstruction

## Abstract

Homografts conduits has been the gold standard for right ventricle to pulmonary artery (RV-PA) conduit. Several different types of xenograft valved conduits have been used as an alternative to homografts, due to their limited availability. In this single center, retrospective study, we analyzed the outcomes of patients with bovine jugular vein conduit (Contegra) and porcine valved conduit (Biointegral) in terms of survival and reintervention rate. Between 2012 and 2023, 44 children underwent surgical repair with RV-PA conduits using Contegra (*n* = 20) or Biointegral (*n* = 24). Patients with truncus arteriosus and patients who underwent unifocalization and Ross procedures were excluded. The operations in which other RV-PA conduits such as homografts, Gore-Tex grafts with PTFE handmade valved were used, were also excluded from the study. The median age of the patients was 19 (3–60) months and 84% of the patients (*n* = 37) had a history of previous intervention. Hospital mortality was 4.5% (*n* = 2). The median length of stay in intensive care unit and hospital was 5 (2–63) and 19 (2–145) days, respectively. 36 of the patients (82%) were followed for a median of 68 (4.8–143.7) months. There was one late death in Contegra group and five late deaths in Biointegral group. Survival analysis revealed that 1, 5, and 10-year survival rates were 100%, 90%, and 90% in Contegra group and 81%, 76.2%, and 33.9% (*p* = 0.047) in Biointegral group, respectively. During follow-up period, 11 patients (30.5%) needed reintervention (*n* = 3 in Contegra; *n* = 8 in Biointegral group). Freedom from reintervention rates were 100%, 94.1%, and 47.1% at 1, 5, and 10 years in Contegra group and 100% and 63.3% at 1 and 5 years in Biointegral group, respectively (*p* = 0.024). In this study, the outcomes of Contegra conduits were statistically significantly better than Biointegral conduits. Contegra is still the most valuable alternative to homografts. We believe that the choice of conduit in the first surgery is an important decision that directly affects survival and re-intervention rates.

## Introduction

Right ventricle to pulmonary artery (RV-PA) valved conduits are necessary for right ventricular outflow tract (RVOT) reconstruction of some congenital heart anomalies. Homografts, harvested from the human pulmonary artery have been the gold standard as a RV-PA conduit ever since its inception in 1966 [1, 2]. Several different types of xenograft valved conduits are used as an alternative to homografts due to their limited availability [3, 4]. The most common xenograft conduits are bovine jugular vein (Contegra; Medtronic Inc, Minneapolis, Minn) and porcine (Biointegral; Biointegral Surgical Inc, Ontario, Canada) pulmonary valved conduits. Contegra is a natural valved conduit, treated with glutaraldehide whereas Biointegral has a porcine semilunar valve, implanted in a porcine pericardial tube graft treated with No-React technology [5]. Both conduits are available in a wide range of sizes (Contegra: 12–22 mm; Biointegral 15–31 mm) and their accessibility is the main advantage of them. No currently available valved conduit option is optimal and all patients with a conduit may need reinterventions at multiple times throughout their lifetime, not only due to a lack of growth potential, but also degeneration and calcification [6, 7]. Reinterventions whether surgical or catheter based, may cause mortality or morbidity. Apart from these, conduit failure itself, either conduit stenosis or valvular insufficiency may cause right ventricle dysfunction, dilatation, hypertrophy, tricuspid valve insufficiency, arrhythmias, and even sudden death [8].

Contegra was introduced to the market in 1999. Since then a lot of promising reports about it have been published. Some comparative studies have delineated similar mid- to long-term results for homografts and Contegra. Nevertheless, stenosis in distal anastomosis, aneurysmal dilatation when exposure to high pressure and a high rate of valvular insufficiency are some drawbacks of Contegra [9, 10]. Although porcine valves have been used for years in cardiac surgery in aortic, mitral or tricuspid valve replacements, reports about mid- to long-term performance of Biointegral pulmonary conduits are very limited. No studies to date  have compared Biointegral pulmonary conduit with Contegra or homografts. Glutaraldehyde pretreatment of bioprosthetic heart valves is the major pathogenic factor for calcific degeneration [5]. Several pre-treatment methods are developed to abbreviate degeneration process. No-React process applied to the Biointegral conduit was developed by Biocar, Belo Horizonte, Brazil in 1996, for the purpose of delineating the merits of aldehyde detoxification process in preventing xenograft dystrophic calcification [5]. To date, there has been no convincing evidence for superiority or effectiveness of one method over the others.

In this single-center, retrospective study, we analyzed the outcomes of patients with Contegra or Biointegral pulmonary conduits in terms of survival and freedom from reintervention.

## Materials and Methods

RV-PA conduits were implanted in 50 children during the repair of congenital heart defects between 2012 and 2023. Patients who underwent truncus arteriosus repair, unifocalization, Ross and Yasui procedures were excluded from the study for uniformity of the patients. Data- including medical histories of the patients, preoperative demographic characteristics, operative details, postoperative outcomes, and follow-up information - were retrieved from the institutional database retrospectively. All patients or their families were contacted by telephone and the patient’s history and functional class, as well as the last postoperative examinations such as echocardiography and angiography results, were obtained. Approval from the institutional ethics committee was taken before establishing the study (ATADEK 2023-21/742). RV-PA conduit types were Contegra in 20 cases (40%), Biointegral in 24 cases (48%), handmade ePTFE valved Gore-Tex graft in 3 cases (6%) and homograft in 3 cases (6%). The patients with handmade valved ePTFE conduits and homografts were also excluded from the study. The remaining 44 patients were divided into two groups according to the conduits used (Table 1).

**Table 1:** Demographic parameters of the patients

	Group contegra (*n* = 20)	Group biointegral (*n* = 24)	*p* value
Age (months) (Median;min–max)	19 (3–36)	18.5 (10–60)	0.687
Female sex (%)	6 (30)	10 (41.7)	0.428
TGA-VSD-PS (%)	4 (20)	2 (8.3)	0.387
VSD-PA (%)	16 (80)	22 (91.7)	
Previous intervention (%)	18 (90)	19 (79.2)	0.333
Conduit size (median; min–max)	18 (16–20)	17 (13–21)	0.671
CPB time (mean ± SD)	112 ± 21	141 ± 67	0.311
X Clamp time (mean ± SD)	60 ± 13	80 ± 36	0.069
ICU time (median; min–max)	5.5 (2–36)	4.5 (2–63)	0.600
Hospital time (median; min–max)	19.5 (5–63)	18.5 (2–145)	0.804
Complications (%)	4 (20)	11 (45)	0.075
In-hospital mortality (%)	0 (0)	2 (8.3)	0.191

The median age of the patients was 19 months (3–60 months). The diagnoses of the patients were transposition of the great arteries with ventricular septal defect and pulmonary stenosis (TGA-VSD-PS) in 6 patients (13.6%) and ventricular septal defect with pulmonary atresia (VSD-PA) in 38 patients (86.4%). A history of previous interventions were present in 37 patients (84%)**;** systemic-to-pulmonary (S-P) shunt operation in 26 patients, ductal stenting in 4 patients, both ductal stenting and S-P shunt in 4 patients, pulmonary banding in 2 patients and RVOT stenting in 1 patient.

### Surgical Technique

All operations were performed by means of a midline sternotomy using conventional cardiopulmonary bypass (CPB) with bicaval cannulation, intermittent tepid blood cardioplegia and moderate hypothermia. Any systemic-to-pulmonary artery shunts were ligated immediately after the  initiation of CPB. VSD was closed to direct left ventricular blood flow to the aorta with a Dacron patch. RV-PA connection was reconstructed with a valved conduit. Pulmonary artery reconstruction if needed and distal conduit to pulmonary artery anastomoses were performed after aortic cross clamping while the heart was beating to reduce the ischemic myocardial period. The choice of conduit was  based on the availability and cost.

### Statistical Analysis

Continuous parameters are  presented as median and range (minimum–maximum), while categorical parameters are shown as  counts and percentages. All statistical analyses were performed using IBM SPSS Statistics software package 29.0. The Student’s *t* test was used for comparisons between different groups of normally distributed quantitative variables, and the Mann–Whitney *U* test was used to compare non-normally distributed quantitative variables between groups. Chi-square (*χ*^2^) test was used for the comparison of categorical variables between groups. Kaplan–Meier survival analysis was  performed for the two conduit groups  to assess 1-, 5-, and 10-year survival and freedom of reintervention. A *p-value of * < 0.05 was considered statistically significant.

## Results

In-hospital mortality  occured in two  patients (4.5%). One of these patients underwent extracorporeal membrane oxygenation (ECMO) support due to an inability to wean from CPB. This patient died under ECMO support due to multiorgan failure. The other patient had a prolonged intensive care unit stay, underwent tracheostomy, and died of sepsis.

The mean cardiopulmonary bypass and aortic cross-clamp times were 128 ± 53 minutes and 71 ± 29 minutes, respectively. The median durations of intensive care unit and hospital stays were 5 days (2–63 days) and 19 days (2–145 days), respectively.

Postoperative complications were evaluated, and it was determined that 29 patients (66%) could be discharged without complications. The most common complications were as follows:  four patients (9%) underwent postoperative re-exploration for bleeding; four patients (9%) required ECMO support; two patients (4.5%) required permanent pacemaker implantation; one patient (2.3%) experienced low cardiac output syndrome; and one patient (2.3%) had a postoperative stroke.  Six patients (13.6%)  required prolonged mechanical ventilation support and two patients (4.5%)  underwent tracheostomy. Temporary dialysis was performed in four patients (9%). There was no statistically significant difference between the two groups in terms of postoperative complications (*p* = 0.075) (Table 1).

**Table 2:** Follow-up data

Reoperations/reinterventions	Group Contegra (*n* = 17)	%	Group biointegral (*n* = 19)	%
Reoperations				
Conduit replacement	1	5.9	2	10.5
Reinterventions				
Transcatheter pulmonary valve replacement	1	5.9	2	10.5
Conduit balloon angioplasty	1	5.9	3	15.8
Conduit stenting	0	0	1	5.3

**Fig. 1:**
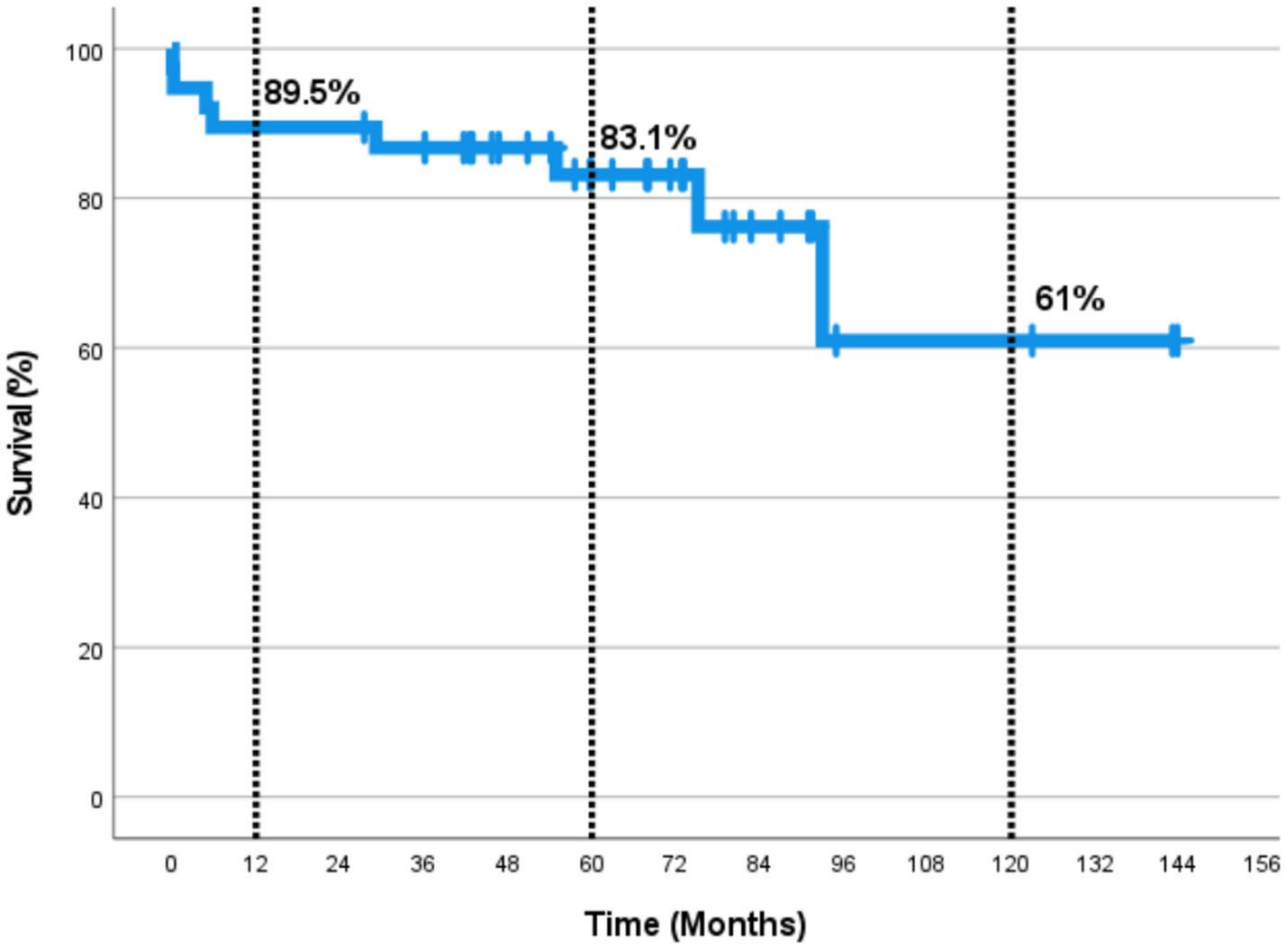
Actuarial survival of all population

Thirty-six patients were followed up  for a median of 68 months (4.8–143.7 months). Six patients were lost to follow-up. There were six late deaths. Three of them were due to sudden cardiac arrest at home. One of these three patients had protein C deficiency and died as a result of acetylsalicylic acid-induced major bleeding. The other two had conduit failure and were awaiting intervention. The remaining three deaths were caused by cerebral ischemia following conduit re-replacement, pneumonia, and ventricular dysfunction. In the Kaplan–Meier survival analysis, the 1-, 5-, and 10-year survival rates for the entire population were 89.5%, 83.1%, and 61%, respectively (Fig. 1).

**Fig. 2:**
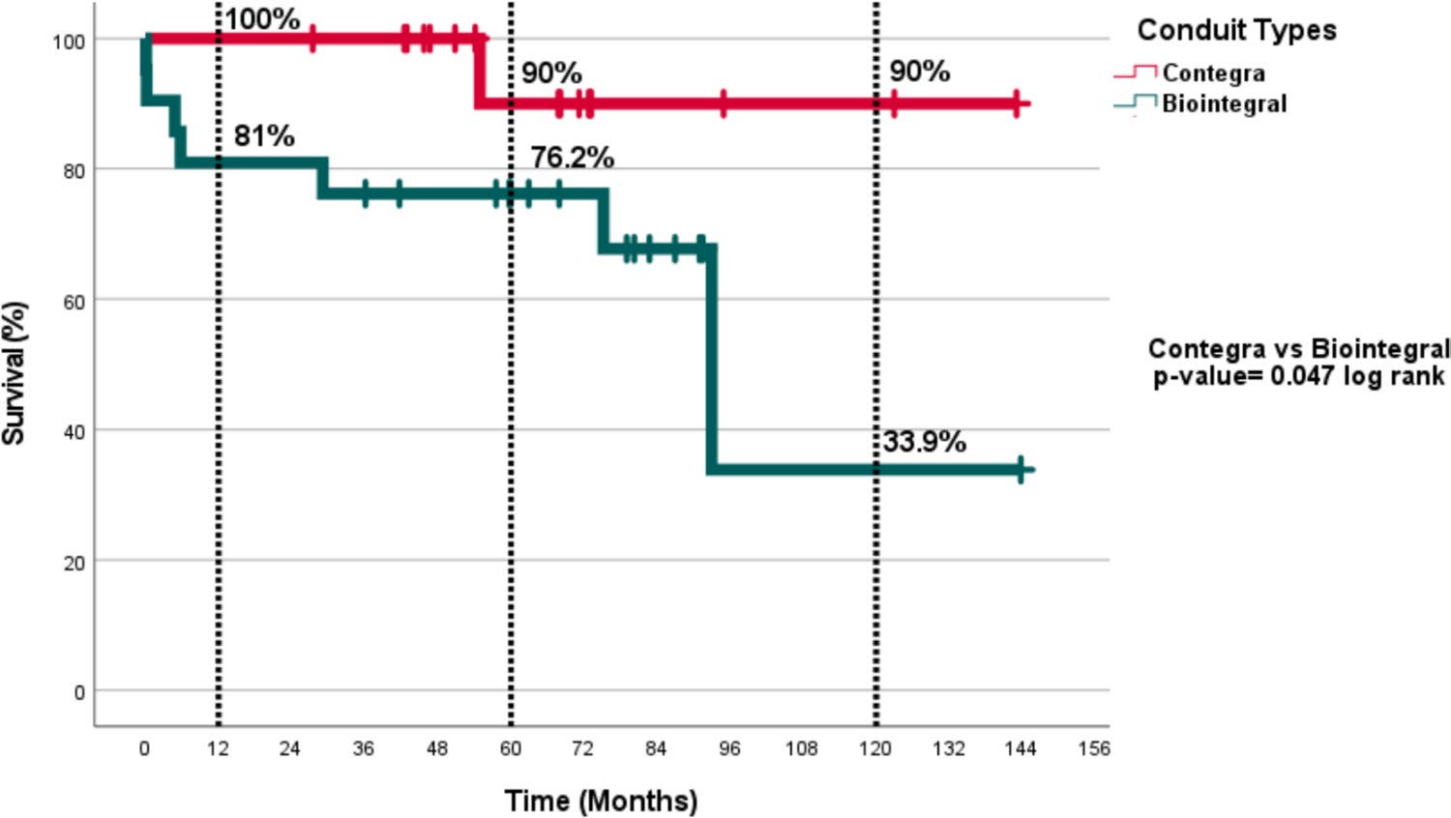
Actuarial survival rates according to the conduit types

The two in-hospital deaths in the entire population  occured in the Biointegral group. There was one late death in the Contegra group and five late deaths in the Biointegral group. According to the Kaplan–Meier survival analysis, 1-, 5-, and 10-year survival rates for the Contegra and Biointegral groups were 100%, 90%, and 90%; and 81%, 76.2%, and 33.9%, respectively (Fig. 2). The difference in survival rates between the groups  was statistically significant (*p* = 0.047).

During the follow-up period, 11 patients (30.5%)  required reintervention. All reinterventions were  related to conduit or right-sided heart problems. The indication for reintervention of the RV-PA conduit was either stenosis or regurgitation  leading to hemodynamic problems, symptoms, or  reduced exercise capacity. Reintervention for stenosis was considered in the presence of any of the following: RV systolic pressure > 60–80 mmHg; progressive RV systolic dysfunction; RV/LV systolic pressure ratio > 0.66; RVOT gradient > 25 mmHg; decreased exercise capacity; or  significant arrhythmias. Reintervention for regurgitation was considered in cases of significant pulmonary regurgitation with clinical symptoms (New York Heart Association [NYHA] class III or IV). In asymptomatic patients, reintervention  was indicated by progressive right ventricle dilatation or dysfunction, new-onset arrythmias and the developement or progression of tricuspid valve regurgitation. If there is a risk for coronary compression in percutaneous pulmonary valve replacement or if conduit stenosis can not be relieved by high-pressure balloon angioplasty and subsequent stenting, then surgical reoperation is considered.

**Fig. 3:**
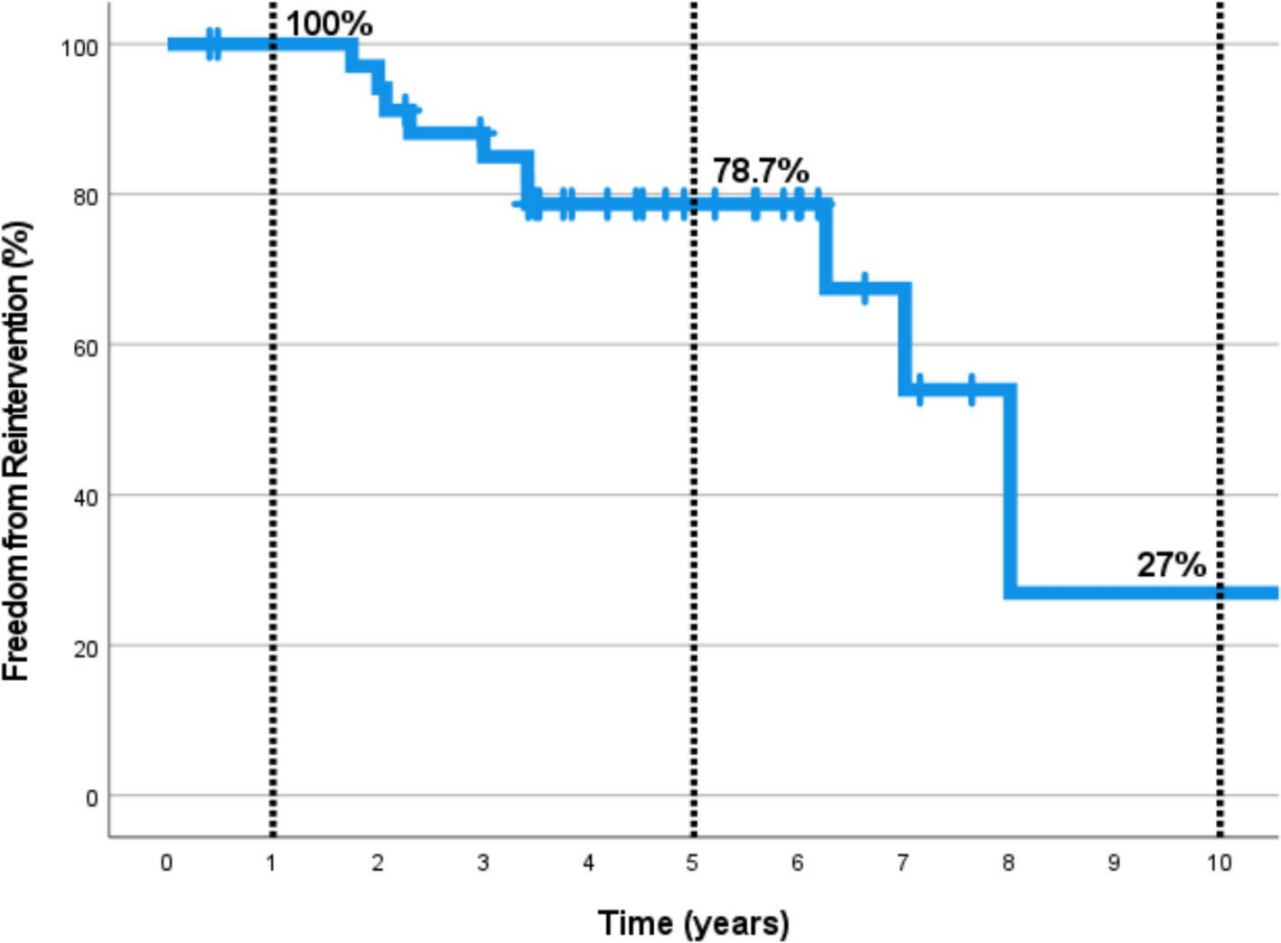
Actuarial freedom from reintervention of all patients

Three patients (8.3%) underwent conduit replacement while four patients (11%)  underwent balloon angioplasty for the RV-PA conduit; three patients (8.3%) underwent transcatheter pulmonary valve replacement (TPVR), and one patient (2.7%) had conduit stenting. The procedures of reoperation and reintervention are summarized in Table 2. In the Kaplan–Meier analysis of freedom from reintervention , the 1-, 5-, and 10-year freedom from reintervention rates for the entire population were 100%, 78.7% and 27%, respectively (Fig. 3).

**Fig. 4:**
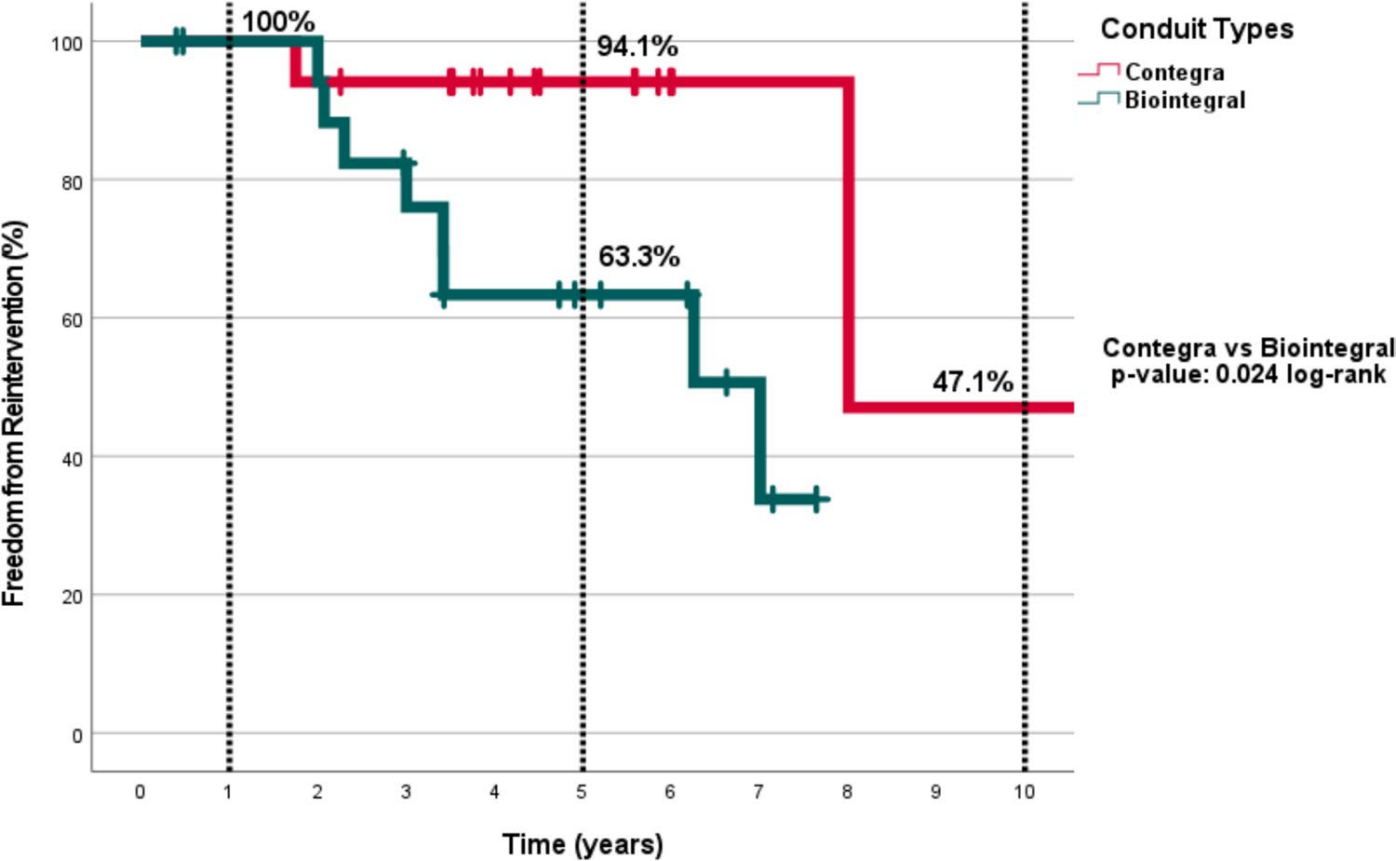
Actuarial freedom from reinterventions according to the conduit types

In the comparative analysis, the 1-, 5-, and 10-year freedom from reintervention rates in the Contegra group were 100%, 94.1%, and 47.1%, respectively.  In the Biointegral group, the 1- and 5-year freedom from reintervention rates were 100% and 63.3%, respectively. The difference between groups were statistically significant (*p* = 0.024) (Fig. 4).

## Discussion

Conduit choice may be the mainstay of a surgical procedure during correction of some congenital heart defects. As conduit failure is inevitable for all kinds of available conduits, all children deserve to have a conduit with the best outcomes. Pulmonary homograft conduit is considered the best for RVOT reconstruction with an approximate 10-year freedom from reoperation of 60–90%, but their scarcity prevents common usage [4, 6, 11]. Studies to develop  an “ideal” conduit are still going on. Different materials, treatment methods, decellularization, and scaffold repopulation are being explored to achieve the ultimate goal [12]. To date, all conduit types are far from being optimal. In this study, we used two different types of xenograft conduits and our results are surprising. First of all, conduit failure is not a “benign” phenomenon. We had six late deaths; half of them died during reintervention and the other half died while waiting for reintervention. Five of the deaths were in the Biointegral group and both actuarial survival and freedom from reintervention rates were statistically different between the groups, favoring the Contegra conduit.

As mentioned before, no comparative study has been performed between Contegra and Biointegral conduits. To design a comparative study is not easy and may have some drawbacks. For example, neonates and infants with small conduits may need replacement earlier. The anatomic position of the conduit may affect longevity. Conduits after the Ross operation may perform better than conduits  that are in an extra-anatomic position after the Rastelli operation. Patients who need unifocalization due to major aorto-pulmonary collaterals may have the worst conduit performance because of the high incidence of underdeveloped pulmonary vascular bed and higher pulmonary vascular resistance [11, 12]. To  eliminate these biases, we excluded those patients from the study so that we could obtain well-balanced study groups. We think that our results may be noteworthy. We  stopped using Biointegral pulmonary conduits and tried to find other alternatives. Handmade ePTFE valved Gore-Tex grafts may be promising with longer durability and lower risk of compression. In our study handmade trileaflet ePTFE valved conduits were sewn as described in Ando et al.’s [13] article. They used these conduits in 139 patients with complex congenital disease in their report. This technique is easy to apply and the grafts are not expensive and are mostly available. Also, as Contegra conduits don’t have  larger sizes, in older children these conduits may be an alternative. Fujita et al. [14] reported that among 502 patients with ePTFE valved conduits, freedom from conduit explantation was 99.5% at 5 years and 89% at 10 years.

Shelhigh pulmonic valved conduit (Shelhigh Inc., Millburn, NJ) is another porcine conduit, harvested unbloc from porcine pulmonary artery. It is a natural conduit and does not contain any suture lines, like Biointegral conduit. According to a comparative study performed by Schoenhoff et al. [15], Contegra performed better than the Shelhigh conduit, although  the difference was not statistically significant. Among 84 patients and 100 implantations (Contegra: 43 and Shelhigh: 57), the mean time to replacement was 18 ± 9 months for the Shelhigh pulmonic graft versus 42 ± 4 months for the Contegra conduit (*p* = 0.25).

Breymann et al. [16] presented  the European Contegra Multicentre Study involving 165 Contegra implants and concluded that results at  seven years were comparable to those of homografts. At  5 years of follow-up, freedom from any event was 13% for infants, 58% for 1–10-year-olds, and 82% for those above 10 years. Protopapas and Athanasiou reviewed the published literature and analyzed midterm outcomes from 17 reports covering a total of 767 patients. After a total follow-up of 573 patient-years, conduit stenosis was observed in 10.9% of the patients, more often in smaller sizes [17].

Brown et al. [10] analyzed their 216 patients who received Contegra (*n* = 153) and pulmonary homografts (*n* = 63). No significant differences were present between the groups with respect to mean age, body surface area, conduit indication or conduit diameter, although the mean follow-up duration was longer in patients  who received homografts. Conduit dysfunction, conduit failure and the need for explantation were more in homografts, albeit at longer follow-up interval.  The rate of conduit dysfunction was significantly higher in the pulmonary homograft cohort (73% and 47% at five and ten years, respectively) compared to the Contegra group (90% and 82% at five and ten years), respectively (*p* < 0.001). Similar to Brown’s series, Hickey et al. [18] compared the Contegra conduit with the homografts in 107 infants who survived truncus arteriosus repair, from 17 different institutions. They stated that the propensity-adjusted 3-year freedom from replacement for in-conduit stenosis was 96.4% for the Contegra and 69.8% for the homograft (*p* = 0.05). It seems that Contegra has a comparable performance with homografts, especially in small sizes. According to our experience, we think that the calcification rate of the Contegra conduits may be lower than that of other conduits, including homografts. Transcatheter reinterventions may be easier for the Contegra conduits when needed. Larger-sized stent grafts can be implanted during transcatheter interventions. However, Biointegral conduits are usually resistant to balloon dilatation due to heavy calcification. When we asked to our colleagues who are expert in transcatheter interventions, they confirmed our findings.

The downsides of the Contegra conduit are distal anastomotic stenosis and conduit dilatation especially during exposure to high pressure. In one of our latest articles [9], we detected severe dilatation in 4 (31%) out of 13 patients who underwent truncus arteriosus repair, after a median follow-up of 36 months. Additionally, three (23%) patients had stenosis at the distal anastomosis. In this study, no conduit dilatation or stenosis in at the distal anastomosis was encountered. Probably small pulmonary artery-conduit mismatch and pulmonary hypertension can be reasons for these complications. One of the  largest series comparing porcine valved conduits to homografts belongs to Dearani et al. [19]. They stated that, the durability of the homografts was inferior  to the porcine valved Dacron conduits in the late follow-up of 1095 patients. Their total freedom from reoperation rates were 55.5% and 31.9% at 10 and 20 years, respectively [19].

As patients with conduit failure may be asymptomatic, periodic clinical examinations with echocardiography are mandatory to detect dysfunction and high pressures in the right ventricle. As mentioned before, patients with conduits are at risk for sudden death or other major adverse events. Timely and proper reinterventions are very important to prevent worse outcomes [11, 12, 20].

In conclusion, the outcomes of the Contegra conduit were statistically significantly better than the Biointegral conduits, in terms of both survival and freedom from reintervention. Contegra is still the most valuable alternative to homografts. We believe that the choice of conduit in the first surgery is an important decision that directly affects survival and reintervention rates.

## Data Availability

No datasets were generated or analysed during the current study.
